# Clinical heterogeneity and treatment optimization in anti-KLHL11 encephalitis: two case reports and literature review

**DOI:** 10.3389/fimmu.2026.1729540

**Published:** 2026-06-01

**Authors:** Lin Han, Ying Li, Chuan Li, Lin Li, Dan Yao, Yunfeng Hao, Xuan Zhou, Yuting Dang, Rong Zhang, Lan Gao, Jun Luo, Jiaxin Yang, Ying Du, Wei Zhang

**Affiliations:** 1 Department of Neurology, Tangdu Hospital, Fourth Military Medical University, Xi’an, Shaanxi, China; 2 Xi’an Medical University, Xi’an, Shaanxi, China

**Keywords:** anti-KLHL11 antibodies, autoimmune encephalitis, efgartigimod, immunotherapy, paraneoplastic neurological syndrome, rituximab

## Abstract

**Objective:**

This study aimed to investigate the clinical features, differential treatment responses, and management strategies of encephalitis associated with anti-Kelch-like protein 11 (KLHL11) antibodies.

**Methods:**

We report two cases of anti-KLHL11 encephalitis that improved following immunotherapy and present a literature review of cases published in the last six years.

**Results:**

Case 1 involved a 52-year-old woman who tested positive for serum anti-KLHL11 antibodies at a 1:100 titer. The patient completely recovered following a treatment regimen comprising intravenous immunoglobulin administration at 400 mg/kg/day for 5 days, combined with low-dose rituximab (100 mg, weekly for three consecutive weeks). Extensive tumor screening revealed no underlying malignancies. In Case 2, a 65-year-old man tested positive for anti-KLHL11 antibodies in both serum (titer 1:320) and cerebrospinal fluid (titer 1:100). Initial high-dose intravenous glucocorticoid pulse therapy, starting at 1000 mg dose and tapered by halving the dose every 3 days until discontinuation, combined with low-dose rituximab (100 mg weekly for 3 consecutive weeks), resulted in a suboptimal response, with symptoms recurring after one month. Subsequent administration of efgartigimod (10 mg/kg) led to marked clinical improvement. A testicular ultrasound revealed an epididymal calcification, potentially indicating a burnt-out germ cell tumor.

**Conclusion:**

Anti-KLHL11 encephalitis exhibits considerable clinical heterogeneity. A high serum anti-KLHL11 titer (1:320) was associated with treatment refractoriness, this association is a preliminary observation that requires validation in larger cohorts. Efgartigimod appears to be a promising therapeutic option for paraneoplastic neurological syndrome.

## Introduction

Anti-Kelch-like protein 11 (KLHL11) antibody-associated encephalitis represents a recently identified subtype of autoimmune encephalitis (AE), first characterized in 2019. This condition is classified as a paraneoplastic AE mediated by antibodies targeting intracellular antigens (1). Approximately 70%–80% of affected male patients present with an associated testicular germ cell tumor, such as seminoma (2). Conversely, occurrences in females are uncommon and typically manifest without tumor association, known as a non-paraneoplastic presentation. However, some female cases have been linked to ovarian teratomas or carcinomas (3). Clinical manifestations are diverse and often include ataxia, diplopia, dysarthria, vertigo, tinnitus, and hearing loss. A minority of patients exhibit symptoms of limbic encephalitis, including neuropsychiatric disturbances, cognitive impairment, and seizures (3, 4). Neuroimaging studies, particularly magnetic resonance imaging (MRI), may reveal T2/fluid-attenuated inversion recovery (FLAIR) hyperintensities in the brainstem, cerebellum, or limbic system, as well as hippocampal or generalized cortical atrophy. However, early MRI findings can be unremarkable in some cases (2). The existing diagnostic methods rely on detecting antibodies in both serum and cerebrospinal fluid (CSF), along with comprehensive tumor screening (5). Immunotherapy is the cornerstone of management, encompassing first-line therapies (corticosteroids, intravenous immunoglobulin (IVIG), and plasma exchange) and second-line agents (rituximab and cyclophosphamide) (1, 2). However, considerable challenges persist in clinical management, including heterogeneous treatment responses, unclear relapse mechanisms, and long-term management of tumor surveillance. Herein, we report two representative cases of anti-KLHL11 encephalitis, including one refractory case. By integrating evidence from recent literature, we systematically explored the clinical heterogeneity, potential mechanisms of refractoriness, and individualized treatment strategies for this disease, aiming to provide valuable insights for clinical practice.

## Cases report

### Case 1

A 52-year-old previously healthy woman with a junior high school education presented with one month of neuropsychiatric symptoms, including emotional lability, manifested by alternating mania and depression, self-harm, aggression, and aimless wandering, and two weeks of generalized tonic-clonic seizures (lasting 2–3 min each time), occurring one month after an upper respiratory tract infection. The patient was admitted to our hospital in January 2025. Serum testing confirmed the presence of anti-KLHL11 antibodies (titer 1:100), while CSF analysis for KLHL11 antibodies, routine parameters, biochemistry, India ink stain, and acid-fast bacilli stain were all negative (**Supplementary Materials**). Cranial MRI (**Figure 1**) and 24-h ambulatory electroencephalogram (EEG) yielded unremarkable results (**Supplementary Materials**). Whole-body 18F-fluorodeoxyglucose positron emission tomography-computed tomography (18F-FDG PET-CT) did not reveal any abnormal metabolic foci (**Figure 2**). Peripheral blood flow cytometry analysis indicated the presence of 14.35% CD19^+^ and 14.28% CD20^+^ B-lymphocytes. The Clinical Assessment Scale in Autoimmune Encephalitis (CASE) indicated a score of 4, suggesting a certain level of severity. This was evidenced by 25/30 Mini-Mental State Examination (MMSE) and 19/30 Montreal Cognitive Assessment (MoCA) scores, with notable impairments in memory, orientation, and calculation abilities. Additionally, a Barthel Index score of 50 indicated impaired daily living activities. The patient was diagnosed with anti-KLHL11 antibody encephalitis and received IVIG at 400 mg/kg/day for 5 days, while corticosteroids were withheld due to familial concerns regarding potential side effects. To reduce the risk of relapse and ensure sustained immunomodulation, treatment was subsequently followed by low-dose rituximab (100 mg) weekly for three consecutive weeks. At the 3-month follow-up, the patient demonstrated complete resolution of symptoms and cognitive recovery, with MMSE and MoCA scores of 29/30 and 26/30, respectively, and a Barthel Index score of 100, along with an effective B-cell depletion (CD19^+^: 0.31%, CD20^+^: 0.24%). At the 6-month follow-up post-discharge, the patient remained asymptomatic, she refused to receive repeat cranial imaging and relevant blood examinations.

**Figure 1:**
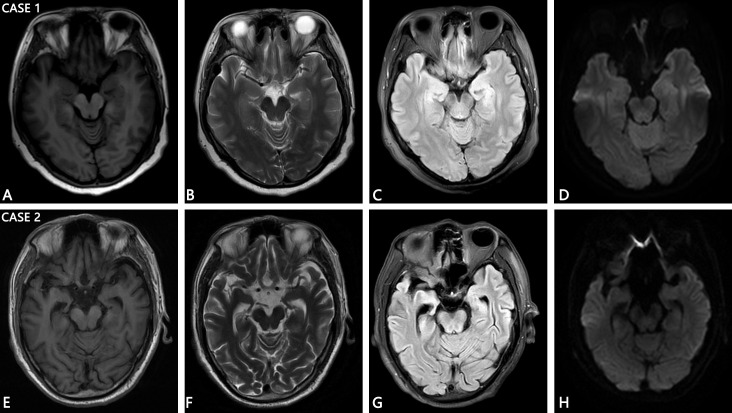
The patients’ cranial MRI during hospitalization. **(A–D)** Case 1: Axial sequences demonstrate unremarkable findings; clinical presentation: neuropsychiatric symptoms and seizures. **(E–H)** Case 2: Axial sequences reveal no signal abnormalities but demonstrate mild bilateral hippocampal and mesial temporal lobe volume losses; clinical presentation: cognitive decline, behavioral disturbance, and limbic dysfunction. Image sequences: **(A, E)** T1-weighted; **(B, F)** T2-weighted; **(C, G)** Fluid-attenuated inversion recovery (FLAIR); **(D, H)** diffusion-weighted imaging (DWI).

**Figure 2:**
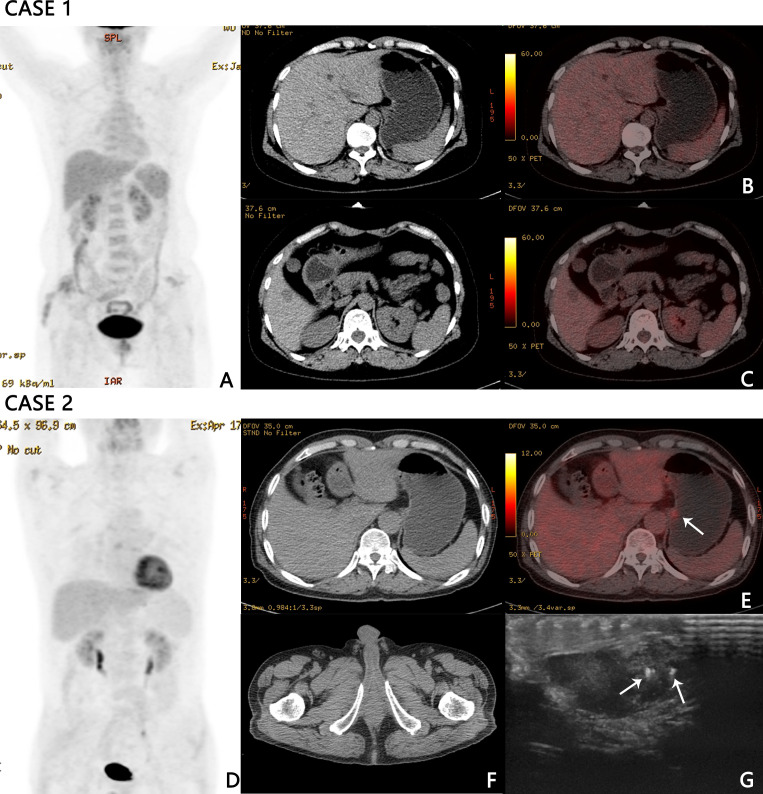
Whole-body 18F-FDG PET-CT imaging findings. **(A–C)** Case 1: **(A)** Maximum Intensity Projection (MIP) image (thorax, abdomen, and pelvis) demonstrates physiologic radiotracer biodistribution without evidence of abnormal hypermetabolic foci. **(B)** Axial PET/CT fusion image (upper abdomen). **(C)** Axial PET/CT fusion image (lower abdomen). **(D–G)** Case 2: **(D)** Maximum Intensity Projection (MIP) image (thorax, abdomen and pelvis) demonstrates physiologic radiotracer biodistribution; no definite abnormal hypermetabolic foci are identified. **(E)** Axial PET/CT fusion image (upper abdomen) exhibits slightly increased glucose metabolism in the gastric cardia (white arrow). **(F)** Axial CT image (genitourinary tract, including testes and epididymides) reveals no significant abnormalities. **(G)** Scrotal ultrasound identifies a calcification within the right epididymis (white arrow).

### Case 2

A 65-year-old male patient with a junior high school education, who had previously undergone laparoscopic cardia-constriction surgery for gastroesophageal reflux disease (now fully resolved) and had no other significant medical history, was admitted due to “progressive unresponsiveness and behavioral abnormalities over 3 months.” In December 2024, he exhibited unexplained cognitive and behavioral disturbances characterized by slowed responsiveness, fecal and urinary incontinence, clothing apraxia, and disorientation. During the course of the illness, he experienced a generalized tonic-clonic seizure with loss of consciousness lasting approximately 10 min, alongside a weight loss of 5 kg. Upon admission, routine CSF parameters were normal; however, CSF biochemistry revealed slightly elevated protein levels (485.6 mg/L). Anti-KLHL11 antibody titers were measured at 1:100 in the CSF and 1:320 in the serum (**Supplementary Materials**). Cranial MRI revealed mild bilateral hippocampal and mesial temporal lobe volume losses (**Figure 1**), and a 24-h EEG indicated no epileptiform activity (**Supplementary Materials**). A whole-body 18F-FDG-PET-CT scan indicated mild glucose hypermetabolism at the cardia, although subsequent esophagogastroduodenoscopy revealed no neoplastic lesions, and imaging identified no other abnormal metabolic foci (**Figure 2**). However, testicular ultrasound revealed a calcified lesion in the right epididymal tail (**Figure 2**), potentially representing an immunologically cleared (regressed/burned-out) germ cell tumor based on prior literature (2, 6). Peripheral blood CD19^+^/CD20^+^ B-lymphocyte counts were recorded at 12.59% and 12.58%, respectively. The cognitive assessment was constrained, with an incomplete MMSE, a CASE score of 8, and a Barthel Index for Activities of Daily Living score of 40. A diagnosis of anti-KLHL11 antibody encephalitis was established. Initial treatment involved high-dose intravenous methylprednisolone (1,000 mg daily, with dosage halved every 3 days until discontinuation), followed sequentially by low-dose rituximab (100 mg weekly for three consecutive weeks) aimed at relapse prevention and long-term immunomodulation. One month after treatment, the patient experienced an exacerbation of psychiatric symptoms, characterized by marked aggression and agitation, with quetiapine proving ineffective. Upon re-admission, profound B-cell depletion was observed (CD19^+^/CD20^+^ counts: 0.46%/0.32%). Attempts to manage symptoms with dexmedetomidine, phenobarbital, and diazepam were unsuccessful. Subsequent administration of intramuscular haloperidol resulted in mutism and lower limb weakness, rendering the patient non-ambulatory by day 3, necessitating the discontinuation of the antipsychotic. Immunotherapy was reinitiated with intravenous efgartigimod alfa (10 mg/kg weekly for 4 weeks). Following this regimen, at the 3-month follow-up post-discharge, the patient’s psychiatric symptoms resolved completely, and cognition significantly improved (MMSE: 27, MoCA: 22, Barthel Index: 90). At the 6-month and 1-year follow-up assessments, the patient demonstrated a favorable clinical recovery, with no indications of disease recurrence. Serial cranial imaging showed no significant progression of cerebral atrophy, no abnormal intracranial signals, and no evidence of tumor lesions (**Supplementary Materials**).

Both patients met the 2016 diagnostic criteria for AE as established by Graus et al. (6). Case 1 was classified as clinically probable AE, supported by characteristic clinical manifestations, the presence of anti-KLHL11 antibodies, and a satisfactory response to immunomodulatory therapy. In contrast, Case 2 was diagnosed with definite AE.

## Methods

CSF samples from both patients were analyzed for anti-KLHL11 antibodies using an indirect immunofluorescence assay based on transfected cells, known as a cell-based assay.

A literature search was conducted in PubMed spanning from January 2019 to May 2026, utilizing the following keywords: “KLHL11 encephalitis,” “anti-KLHL11 antibody,” and “paraneoplastic encephalitis.” The inclusion criteria encompassed published, peer-reviewed cases with confirmed anti-KLHL11 antibodies, along with documented clinical phenotypes and treatment details. Studies were excluded if they were abstracts without full text or involved non-human subjects. Following the literature screening process, a total of 11 studies met the eligibility criteria and were included in the review. A descriptive summary was then compiled, detailing the clinical features, tumor associations, treatment responses, and clinical outcomes. It is important to note that this work constitutes a narrative review rather than a systematic review.

## Discussion

### 1 Marked clinical heterogeneity in manifestations

Previous research has predominantly highlighted that KLHL11 antibody-associated encephalitis is characterized by a marked male predominance (> 90%), frequent association with testicular tumors (70%–80%), and cerebellar ataxia as the primary symptom (3). However, the two cases presented in this study, a 52-year-old female and a 65-year-old male, exhibited a notably different profile: Both individuals lacked the classic ataxia symptoms; instead, they presented with psychiatric or behavioral abnormalities or the onset of seizures. The absence of these core symptoms significantly increases the risk of clinical misdiagnosis, potentially leading to incorrect attribution to viral encephalitis, primary psychiatric disorders, or other subtypes of encephalitis. Additionally, there is observed heterogeneity in antibody distribution patterns, with some patients testing negative for serum KLHL11 antibodies (4, 7, 8). In this case report, Case 1 was exclusively serum-positive, whereas Case 2 was both serum- and CSF-positive, with significantly elevated serum titers (1:320) compared to CSF titers (1:100). This finding suggests that the immune activation in Case 2 may be predominantly peripheral, potentially influenced by tumor-associated immune responses. Moreover, neuroimaging results varied between the cases: Case 1 displayed no characteristic abnormalities on brain MRI, while Case 2 demonstrated atrophy in the medial temporal lobe and cerebral cortex. This finding aligns with the existing literature, which reports that approximately 20% of patients may not present with abnormal imaging signals (2). The atypical inflammatory manifestations observed in our cases are likely attributable to the early presentation of the disease. All patients were diagnosed during the initial clinical phase, prior to the development of definitive parenchymal and meningeal pathological lesions. Consequently, typical inflammatory features were absent in imaging and laboratory assessments. In conclusion, atypical features, such as female presentation, absence of ataxia, and CSF antibody negativity, highlight the clinical heterogeneity of KLHL11-antibody encephalitis. This underscores the need for clinicians to remain vigilant. Prompt and comprehensive serum and CSF antibody testing is crucial to avoid delayed or missed diagnoses, thereby facilitating the timely initiation of immunotherapy.

### 2 Mechanisms underlying differential treatment responses and drug selection

Both patients with anti-KLHL11 antibody encephalitis in this case report displayed divergent responses to immunotherapy, clearly illustrating the complexity of the disease’s immunopathogenic mechanisms and underscoring the need for individualized treatment approaches. Previous research has indicated that the pathogenicity of KLHL11 antibodies involves multiple stages: KLHL11 polypeptides activate CD4^+^ T cells and drive cytotoxic responses from CD8^+^ T cells, while B cell-derived KLHL11-IgG further exacerbates T cell-mediated injury (2, 9). As an intracellular antigen, KLHL11 mainly triggers T-cell-mediated cytotoxicity; anti-KLHL11 antibodies further induce targeted immune damage to KLHL11-expressing cells within the cerebellum, hippocampus, brainstem and testicular tumor tissues, ultimately causing neurological dysfunction (3). Nonetheless, persistently high titers of anti-KLHL11 IgG may fuel local inflammatory cascades, sustain antigen presentation, and exacerbate T cell-driven tissue damage. By systemically reducing pathogenic IgG levels, efgartigimod may interrupt these self-amplifying immune loops even when the target antigen is localized intracellularly.

Rituximab, a CD20-targeted monoclonal antibody, has established efficacy in the treatment of AE (10). Differing from B-cell lymphoma with massive B-cell overgrowth, autoimmune encephalitis is mainly mediated by functionally abnormal B cells with normal peripheral quantities (11). Therefore, low-dose rituximab is sufficient to achieve robust depletion of circulating CD20^+^ B cells. In this study, both patients received the low-dose rituximab regimen of 100 mg administered weekly for three consecutive weeks to eliminate pathogenic B cells and block autoantibody generation (12).

In Case 1, characterized by the absence of a tumor and a low antibody titer, sustained remission was successfully achieved following immunotherapy with IVIG, and maintenance therapy with rituximab effectively inhibited the emergence of new antibody-producing cell sources. Conversely, Case 2, which involved a suspected regressed germ cell tumor and a high antibody titer, experienced a relapse despite intensive immunosuppressive treatment, including rituximab. This refractoriness was attributed to several factors, including the presence of highly autonomously activated effector T cells (13); persistent pathogenic antibody production by long-lived plasma cells lacking CD20 expression (14); the maintenance of local T cell responses by other antigen-presenting cells (15); and continuous stimulation by potential tumor antigens (16). Collectively, these mechanisms reduced the effectiveness of rituximab, which indirectly suppresses T cells via CD20^+^ B cell depletion. In this case, the patient exhibited notable and rapid symptomatic improvement following the administration of the FcRn antagonist efgartigimod. The mechanism of action of this antagonist involves the acceleration of the lysosomal catabolic pathway for all IgG subtype antibodies, including pathogenic anti-KLHL11 antibodies, by inhibiting FcRn-IgG interactions (17). Moreover, recent research indicates that efgartigimod not only reduces IgG levels but also promotes B cell differentiation into plasma cells with a regulatory phenotype (18). These newly generated plasma cells may exert protective effects, representing an additional significant mechanism contributing to clinical improvement (19). Compared to traditional immunosuppressants, including mycophenolate mofetil and cyclophosphamide, which are associated with hematologic suppression and increased risk of infection, efgartigimod does not induce broad immunosuppression, thereby offering a favorable safety profile and rapid onset of action (20). This study provides a hypothesis-generating observation of the successful off-label use of efgartigimod in a patient with anti-KLHL11 antibody encephalitis. As this agent was administered off-label and no controlled clinical data exist, the present observation is hypothesis-generating and cannot support definitive therapeutic recommendations; thus, controlled clinical studies are required to confirm its efficacy and safety in anti-KLHL11 encephalitis.

### 3 Clinical management strategies

Long-term Oncological Surveillance: Previous research has demonstrated a significant correlation between KLHL11 antibody encephalitis and testicular germ cell tumors, including regressed seminoma (2, 4, 9). Consequently, conducting long-term surveillance is essential even when initial screening methods, such as PET-CT, yield negative results. For male patients diagnosed with KLHL11 antibody encephalitis, an annual ultrasound examination of the reproductive system, specifically the testes and epididymis, is required. Furthermore, it is recommended to regularly monitor serum tumor markers, including alpha-fetoprotein, beta-human chorionic gonadotropin, and placental alkaline phosphatase, at intervals of 3–6 months for a minimum of 5 years (4, 21). Non-specific lesions detected during surveillance, such as epididymal calcification observed in Case 2, should be closely monitored, as they may indicate “burned out tumors” with potential ongoing immunogenicity, and integrated into the surveillance protocol (9, 22).

Selection of Antipsychotic Agents: Psychiatric and behavioral abnormalities are frequently observed as common or even initial symptoms of this disease. In Case 2, the administration of the potent typical antipsychotic haloperidol resulted in severe drug-induced Parkinsonism, characterized by mutism, muscle rigidity, and akinesia. This case underscores the significant risk associated with the use of such agents in these patients. The underlying mechanism is likely linked to autoimmune inflammation, which may alter dopamine receptor function or sensitivity within the basal ganglia. The subsequent penetration of the drug across the blood-brain barrier further blocks dopaminergic neurotransmission, thereby inducing extrapyramidal symptoms (23). Consequently, typical antipsychotics, such as haloperidol and fluphenazine, should be avoided in patients presenting with psychiatric symptoms. Alternatively, atypical antipsychotics should be prioritized, which have a lower propensity for causing extrapyramidal side effects, such as quetiapine and olanzapine. It is crucial to adhere to the principle of initiating therapy at low doses and titrating slowly. For managing acute agitation, short-term sedation with benzodiazepines, such as lorazepam or diazepam, may be considered. In Case 2, haloperidol was notably administered only after the failure of the previously mentioned alternative agents to manage the psychiatric symptoms. Ultimately, the underlying immunotherapy with efgartigimod concurrently ameliorated these psychiatric manifestations. In addition, patients with new-onset neuropsychiatric symptoms, especially atypical or treatment-refractory cases, should be tested for a broad panel of AE antibodies (including anti−KLHL11) to avoid misdiagnosis as primary psychiatric disorder.

Biomarker-guided Personalized Immunotherapy: The intensity and duration of maintenance immunotherapy should be tailored to the individual patient and informed by dynamic biomarker assessments. Regular monitoring of the proportion of peripheral blood CD19^+^/CD20^+^ B cells, ideally conducted every 3–6 months, is critical to achieve sustained depletion to very low levels (< 1%) (24), This parameter serves as a critical indicator for preventing B-cell-mediated disease relapse in patients receiving rituximab therapy. Additionally, serial assessments of serum anti-KLHL11 antibody titers are essential. Effective treatment should result in a significant decline of over 50% or seroconversion to negative status. Variations in serum antibody titers may reflect not only T cell-mediated immune responses but also B cell-related immune mechanisms (25). Persistently elevated or increasing titers indicate ongoing plasma cell activity and an increased risk of relapse, thereby necessitating the intensification or modification of the immunotherapeutic regimen.

However, this study has several limitations. First, it is a small case series with limited follow-up, restricting the generalizability of findings. Second, the literature review is non-systematic and narrative in design. Third, no *in vitro* mechanistic validation was performed to confirm our hypotheses. Finally, efgartigimod was used off-label without controlled trial data, preventing definitive therapeutic conclusions. Larger cohorts, longer follow-up, and mechanistic studies are needed to validate our observations.

## Conclusion

This case report highlights the significant clinical and immunological variability observed in anti-KLHL11 antibody encephalitis. The prognosis is closely linked to antibody titers and tumor status. Patients without tumors and with low serum antibody titers typically respond well to standard immunosuppressive treatments combined with B-cell-targeted therapies. Conversely, elevated serum titers (> 1:100) or serum titers exceeding CSF levels may indicate a refractory disease course and ongoing plasma cell activation, requiring a stratified therapeutic approach. This study provides a hypothesis-generating observation supporting the potential efficacy of efgartigimod in refractory anti-KLHL11 antibody encephalitis. Furthermore, male patients require prolonged surveillance (≥ 5 years), involving serial genital ultrasound and tumor marker assessments. Customizing treatment based on antibody profiles and diligent tumor monitoring is essential for optimizing patient outcomes.

## Data Availability

The raw data supporting the conclusions of this article will be made available by the authors, without undue reservation.
