# Supplementary material for: Clinical heterogeneity and treatment optimization in anti-KLHL11 encephalitis: two case reports and literature review
Source: Front Immunol. 2026 Jun 1;17:1729540. doi: 10.3389/fimmu.2026.1729540 (PMC13265456; doi:10.3389/fimmu.2026.1729540)
Supplement: Supplementary file 1 [file DataSheet1.pdf]

CARE case report guidelines

CARE Checklist of information to include when writing a case report

(cc) BY-NC-ND

Topic	Item	Checklist item description	Reported on Line
Title	1	The diagnosis or intervention of primary focus followed by the words "case report"	P1
Key Words	2	2 to 5 key words that identify diagnoses or interventions in this case report, including "case report"	P2
Abstract (no references)	3a	Introduction: What is unique about this case and what does it add to the scientific literature?	P2
	3b	Main symptoms and/or important clinical findings	P2
	3c	The main diagnoses, therapeutic interventions, and outcomes	P2
	3d	Conclusion—What is the main "take-away" lesson(s) from this case?	P2
Introduction	4	One or two paragraphs summarizing why this case is unique (**may include references**)	P2, P3
Patient Information	5a	De-identified patient specific information.	P3, P4
	5b	Primary concerns and symptoms of the patient.	P3, P4
	5c	Medical, family, and psycho-social history including relevant genetic information	P3, P4
	5d	Relevant past interventions with outcomes	P3, P4
Clinical Findings	6	Describe significant physical examination (PE) and important clinical findings.	P3, P4
Timeline	7	Historical and current information from this episode of care organized as a timeline	P3, P4
Diagnostic Assessment	8a	Diagnostic testing (such as PE, laboratory testing, imaging, surveys).	P3, P4
	8b	Diagnostic challenges (such as access to testing, financial, or cultural)	
	8c	Diagnosis (including other diagnoses considered)	P3, P4
	8d	Prognosis (such as staging in oncology) where applicable	P3, P4
Therapeutic Intervention	9a	Types of therapeutic intervention (such as pharmacologic, surgical, preventive, self-care)	P3, P4
	9b	Administration of therapeutic intervention (such as dosage, strength, duration)	P3, P4
	9c	Changes in therapeutic intervention (with rationale)	P3, P4
Follow-up and Outcomes	10a	Clinician and patient-assessed outcomes (if available)	P3, P4
	10b	Important follow-up diagnostic and other test results	P3, P4
	10c	Intervention adherence and tolerability (How was this assessed?)	P3, P4
	10d	Adverse and unanticipated events	P3, P4
Discussion	11a	A scientific discussion of the strengths AND limitations associated with this case report	P5, P6
	11b	Discussion of the relevant medical literature **with references**.	P5, P6
	11c	The scientific rationale for any conclusions (including assessment of possible causes)	P5, P6
	11d	The primary "take-away" lessons of this case report (without references) in a one paragraph conclusion	P7
Patient Perspective	12	The patient should share their perspective in one to two paragraphs on the treatment(s) they received	P3, P4
Informed Consent	13	Did the patient give informed consent? Please provide if requested	Yes ☑ No ☐
