# Supplementary material for: Clinical heterogeneity and treatment optimization in anti-KLHL11 encephalitis: two case reports and literature review
Source: Front Immunol. 2026 Jun 1;17:1729540. doi: 10.3389/fimmu.2026.1729540 (PMC13265456; doi:10.3389/fimmu.2026.1729540)
Supplement: Supplementary file 2 [file DataSheet2.pdf]

Table 1. Patients Clinical Characteristics and Diagnostic Findings.

Category	Admission		Reference/Notes
	Case 1	Case 2	
**Characteristics**			
Age (year)	52	65	
sex	female	male	
**Laboratory variables**			
Hemoglobin (g/L)	115	146	
fasting glucose (mmol/L)	5.24	4.85	
liver function	normal	normal	
kidney function	normal	normal	
thyroid function	normal	normal	
infectious disease markers (HIV, syphilis, hepatitis)	normal	normal	
CSF white blood cell count ($\times 10^6$/L)	4	0	0-5
CSF protein level (mg/L)	435.2	485.6	150-450
CSF glucose (mmol/L)	3.91	3.0	2.5-4.5
CSF chloride (mmol/L)	129.7	124.4	120-130
CSF-OCBs	negative	negative	negative
serum immunoglobulin G (g/L)	7.95	16.7	7-16
CSF immunoglobulin G	11.3	25.4	0-34
CSF-albumin	248	334	0-350
Anti-KLHL11 antibody titers (CSF)	-	1:100	
Anti-KLHL11 antibody titers (serum)	1:100	1:320	
blood B cell (CD19+/CD20+)	14.35%/14.28%	12.59%/12.58%	
**score of Symptoms**			
MMSE	25	poor cooperation	30
MoCA	19	poor cooperation	30
Barthel Index score	50	40	100

Abbreviations: CSF, cerebrospinal fluid; OCBs, oligoclonal bands; MMSE, Mini-Mental State Examination; MoCA, Montreal Cognitive Assessment.

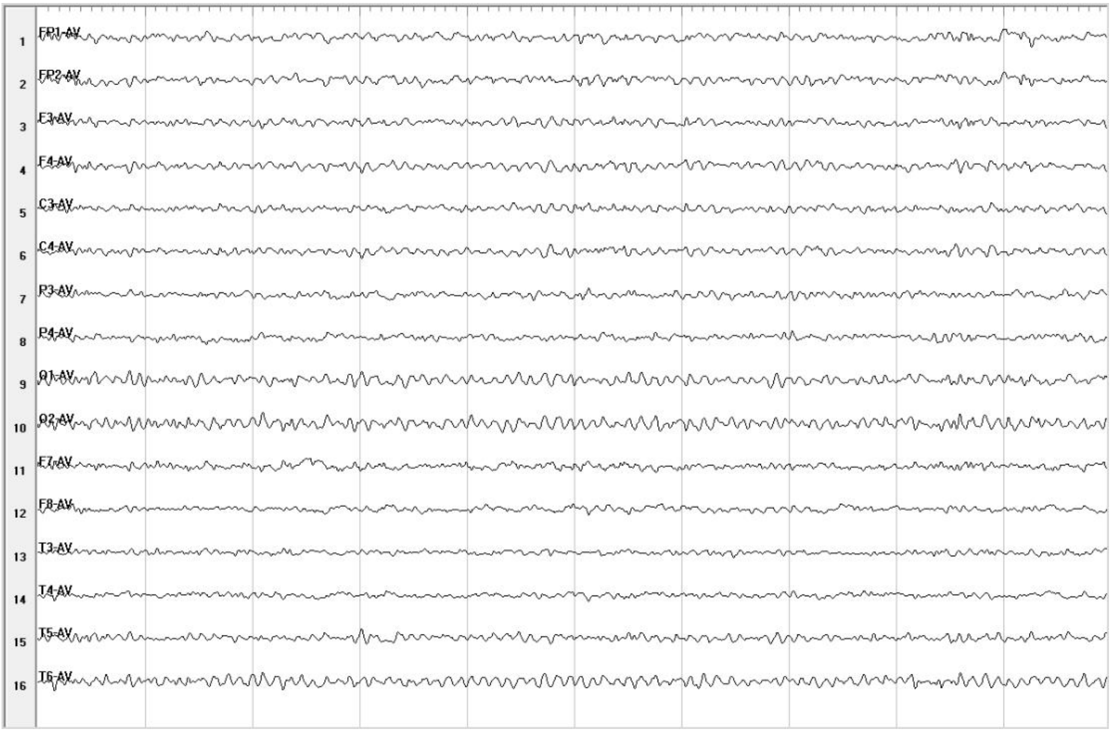

Figure 3. No abnormal waveforms were identified on electroencephalography (EEG) recordings in Case 1.

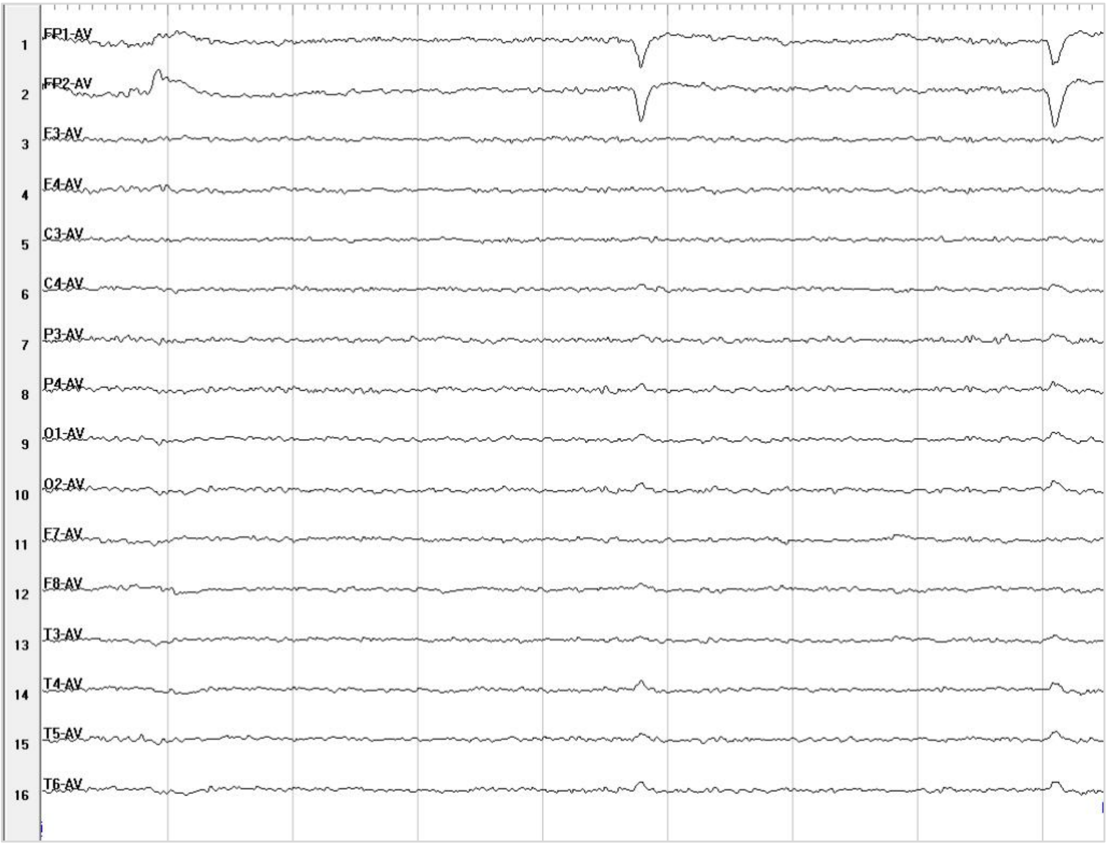

Figure 4. No abnormal waveforms were identified on electroencephalography (EEG) recordings in Case 2.

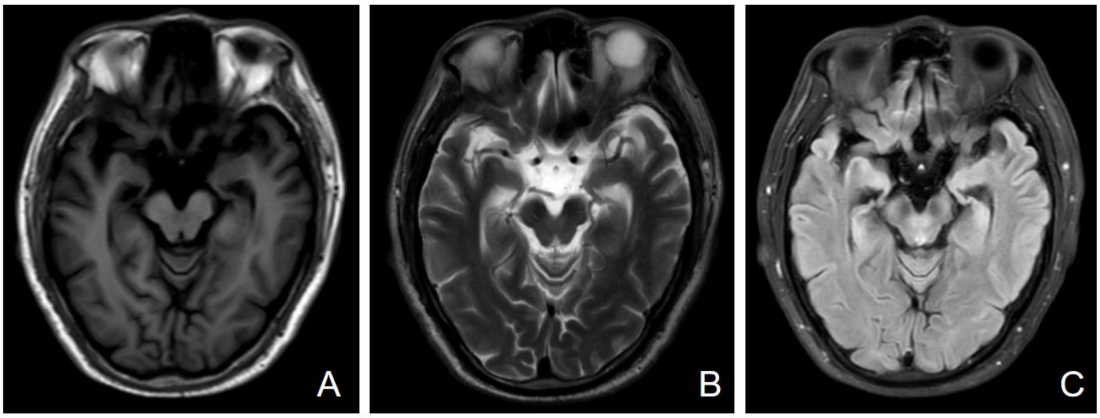

Figure 5. At the 1-year post-discharge follow-up, cranial MRI in Case 2 showed no significant changes compared with previous examinations. Axial sequences: (A) T1-weighted, (B) T2-weighted, (C) Fluid-attenuated inversion recovery (FLAIR). No new abnormal signals or lesions were detected.
